# “It all needs to be a full jigsaw, not just bits”: exploration of healthcare professionals’ beliefs towards supported self-management for long-term conditions

**DOI:** 10.1186/s40359-019-0319-7

**Published:** 2019-06-24

**Authors:** Niall Anderson, Gozde Ozakinci

**Affiliations:** 1 Public Health Department, NHS Borders, Melrose, TD6 9BD UK; 2 School of Medicine, University of St Andrews, St Andrews, KY16 9TF UK

**Keywords:** Long-term, Physical, Condition, Supported, Self-management, Healthcare, Health, Belief, Intention

## Abstract

**Background:**

*Long-Term Conditions* are physical health issues which profoundly impact physical and psychological outcomes and have reached epidemic worldwide levels. An increasing evidence-base has developed for utilizing *Supported Self-Management* to ensure *Health, Social Care & Voluntary* staff are knowledgeable, skilled, and experienced to enable patients to have the confidence and capability to self-manage their conditions. However, despite Health Psychology theories underpinning chronic care models demonstrating beliefs are crucially associated with intention and behaviour, staff beliefs towards *Supported Self-Management* have received little attention. Therefore, the study aimed to explore healthcare professionals’ beliefs towards *Supported Self-Management* for *Long-Term Conditions* using the *Theory of Planned Behaviour*.

**Methods:**

A mixed-methods approach was conducted within a single UK local government authority region in 2 phases: (1) Qualitative focus group of existing *Supported Self-Management* project staff (*N* = 6); (2) Quantitative online questionnaire of general *Long-Term Conditions* staff (*N* = 58).

**Results:**

(1) Eighty two utterances over 20 theme sub-codes demonstrated beliefs that *Supported Self-Management* improves healthcare outcomes, but requires enhancements to patient and senior stakeholder buy-in, healthcare culture-specific tailoring, and organizational policy and resources; (2) Mean scores indicated moderate-strength beliefs that *Supported Self-Management* achieves positive healthcare outcomes, but weak-strength intentions to implement *Supported Self-Management* and beliefs it is socially normative and perceived control over implementing it. Crucially, regression analyses demonstrated intentions to implement *Supported Self-Management* were only associated with beliefs that important others supported it and perceived control over, or by whether it was socially encouraged.

**Conclusions:**

Healthcare professionals demonstrated positive attitudes towards *Supported Self-Management* improving healthcare outcomes. However, intentions towards implementing this approach were low with staff only slightly believing important others (including patients and clinicians) supported it and that they had control over using it. Future *Supported Self-Management* projects should seek to enhance intention (and consequently behaviour) through targeting beliefs that important others do indeed actually support this approach and that staff have control over implementing it, as well as enhancing social encouragement.

**Electronic supplementary material:**

The online version of this article (10.1186/s40359-019-0319-7) contains supplementary material, which is available to authorized users.

## Background

### Long-term conditions

*Long-term Conditions* (LTCs), also termed *Chronic Conditions* [1, 2], comprise complex physical health issues which require ongoing specialist support to enable patients to live with the permanent and/or disabling effects of conditions [3–5]. LTCs encompass a range of conditions which may be treated but are infrequently cured and vary in prevalence, severity, and consequences [6]. LTCs are the leading cause of premature and preventable mortality worldwide, with no country yet successfully reducing LTC levels [2, 7]. Therefore, a significant challenge is faced to target the negative impact of LTCs on life expectancy, healthy life expectancy, healthcare utilisation and expenditure, long-term sickness absence costs, disability, and the likelihood of experiencing co-morbid physical health conditions [2, 8–12]. In addition to physical and medical effects, LTCs are associated with increased risk of depressive or anxiety disorders, which may have profound negative implications for self-care, symptom severity, medication adherence, health behaviours, and LTC-related relapse and survival [3, 13–19].

In addition to the medical, physical and psychological impact of individual LTCs [3], patients may also experience multi-morbid conditions where several LTCs co-exist or co-morbid conditions where several LTCs stem from one *Index* LTC [20, 21]. 33% UK adults and 50% of over 60 year-olds experience at least one LTC, with two or more *Multi-Morbid* conditions present in 65% 65–84 year-olds and 82% over 85 year-olds [6, 22, 23]. As a consequence, LTC patients’ require 66% NHS England expenditure, 50% GP and 64% outpatient appointments, and 70% acute care and inpatient bed stays [23, 24]. Furthermore, as LTCs also have profound mental health consequences [3], 9% NHS England expenditure is required specifically for the psychological impact of LTCs [13–15, 17, 19, 25, 26]. Crucially, the challenges posed by LTCs are mirrored globally with similar epidemic levels and increases across all age groups experienced worldwide [22, 27–30]. Therefore, a movement towards a proactive, collaborative, person-focused approach supporting people to effectively manage their health is required [6, 31].

### Supported self-management

The *Chronic Care Model* [32] provided an initial framework for the development of a collaborative care approach, which was subsequently supplemented by the *Innovative Care for Chronic Conditions* framework [5, 33] and *Expanded Chronic Care Model* [34]. Despite comprising different components, these models highlighted the importance of evidence-based system design, organizational and community support, and patient-professional interactions to support self-management [1]. As a consequence, *Person-Centred Care* (PCC) approaches were developed which promote personalised, coordinated, enabling and respectful healthcare to support patients to have the knowledge, skills and confidence to make informed decisions about their condition(s) and treatment(s) [35]. While intuitively a common-sense approach which *Health, Social Care & Voluntary* (HSV) individuals may assume is already provided, there is not a universally accepted model of PCC implementation and PCC is not routinely conducted despite being central to healthcare policies in the UK and beyond [35–37].

The UK-based charity The Health Foundation’s PCC review [35] outlines multiple healthcare implementation approaches, including collaborative care and support planning, experience-based co-design, person- and family-centred care, and shared decision making. However *Supported Self-Management* (SSM), also termed *Coordinated* or *Integrated Care*, is the most increasingly promoted and implemented PCC approach which builds upon self-care and self-management [35]. Self-care includes behaviours conducted to reduce health-impairing, and enhance health-promoting, behaviours [38], while self-management categorises taking responsibility to proactively manage condition(s) and treatment(s) [39]. SSM enhances these approaches to promote HSV knowledge, skills, experience, confidence and support to ensure patients are supported to effectively self-manage health and overcome social, personal, environmental and economic LTC challenges [6, 39].

An increasing evidence-base is emerging for SSM implementation in both generic and specific LTC settings [35]. Whole-system LTC SSM approaches include Alaska’s *Nuka System of C*are, Germany’s *Proactive Chronic Care Management Program*, Netherland’s *Buurtzorg Model*, Florida’s *A Healthy State* programme, and Hong Kong’s *Chronic Disease Self-Management Programme*, which enhanced multiple patient, healthcare professional, and organizational outcomes [40–45]. Furthermore, SSM programmes have been a particular focus of diabetic healthcare settings [46, 47], including the UK’s *Year of Care* programme [48] which developed from issues with traditional healthcare methods, patient and HSV feedback, national policies, and theoretical support for the Chronic Care Model [32]. Due to positive outcomes from the diabetes-specific programme, the LTC-general *House of Care Model* was subsequently to support collaborative care planning and processes through enabling patients, HSV staff, organizations and commissioners to promote SSM [6, 49].

SSM programmes have resulted in improvements for patients’ medical, health and preference-based treatment outcomes, HSV engagement, satisfaction and skills, patient-professional communication, activation and shared-decision making, and healthcare costs, utilization and adherence [35, 50–59]. However, despite the evidence-base supporting SSM, if implementation is not appropriately promoted and supported, components intended as facilitators may instead be barriers [46, 60]. These may include patient and professional characteristics (including values, attitudes, knowledge and demographics), patient-professional interactions (including communication styles, discrepancies in understanding, and trust), LTC characteristics and treatments (including symptom presentations, multi-morbidity, and treatment availability), and organizational cultures and infrastructures (including staff availability, SSM-promotion and support) [35]. Crucially, to overcome potential barriers and facilitate the development of evidence-based SSM programmes which effectively facilitate and maintain behaviour change, an understanding of underlying psychological principles is key [24].

### Health, social care & voluntary staff beliefs

Substantial research has explored patient beliefs towards SSM and the systematic facilitators and barriers for HSV and systems to implement SSM [6, 33, 59–75]. However, a fundamental principle of the chronic care models [32–34] from which SSM developed is that specific beliefs are required for a behaviour to occur [2]. Therefore, despite collaboration between patients and HSV staff being critical to whether or not SSM occurs, (to the researchers’ knowledge) research has not assessed the crucial component of HSV beliefs towards SSM [24]. *Health Behaviour Models* seek to determine associations between health beliefs and behaviours [76]. The *Theory of Planned Behaviour* (TPB) is a prominent Health Behaviour Model which (like all models) is not without challenges [77–79], but as a pre-existing, validated TPB questionnaire development guide exists to facilitate the assessment of the likelihood of specific health behaviours occurring, it has provided a framework to assess HSV beliefs towards a range of behaviours including care approach, safety behaviours, hand hygiene, and identification of patients at high clinical risk [80–86].

The TPB proposes that whether one conducts a behaviour is associated with intention towards it. Intention is associated with three key direct beliefs measured through directly asking questions on these constructs, which are each influenced by two indirect beliefs which may be measured through indirectly asking about elements which may tap into direct beliefs [77, 78, 86]. Within the SSM context, *Direct Attitude* relates to whether SSM is perceived to have positive or negative LTC outcomes, which may be influenced by *Indirect Behavioural Beliefs* of perceived positive or negative consequences of SSM such as improvements to patient outcomes, combined with *Indirect Outcome Evaluations* of the perceived desirability of consequences. *Direct Subjective Norms* relates to social pressure to conduct SSM which may be influenced by *Indirect Normative Belief* perceptions of what important others (such as GPs in Primary Care settings) feel about SSM, combined with *Indirect Outcome Evaluation* of the important of their approval. Finally *Direct Perceived Behavioural Control* relates to perceived efficacy to conduct SSM, which may be influenced by *Indirect Control Beliefs* of perceived ability to actually conduct SSM if required, combined with *Indirect Influence of Control* for confidence about doing so.

### Study objectives

**Fig. 1:**
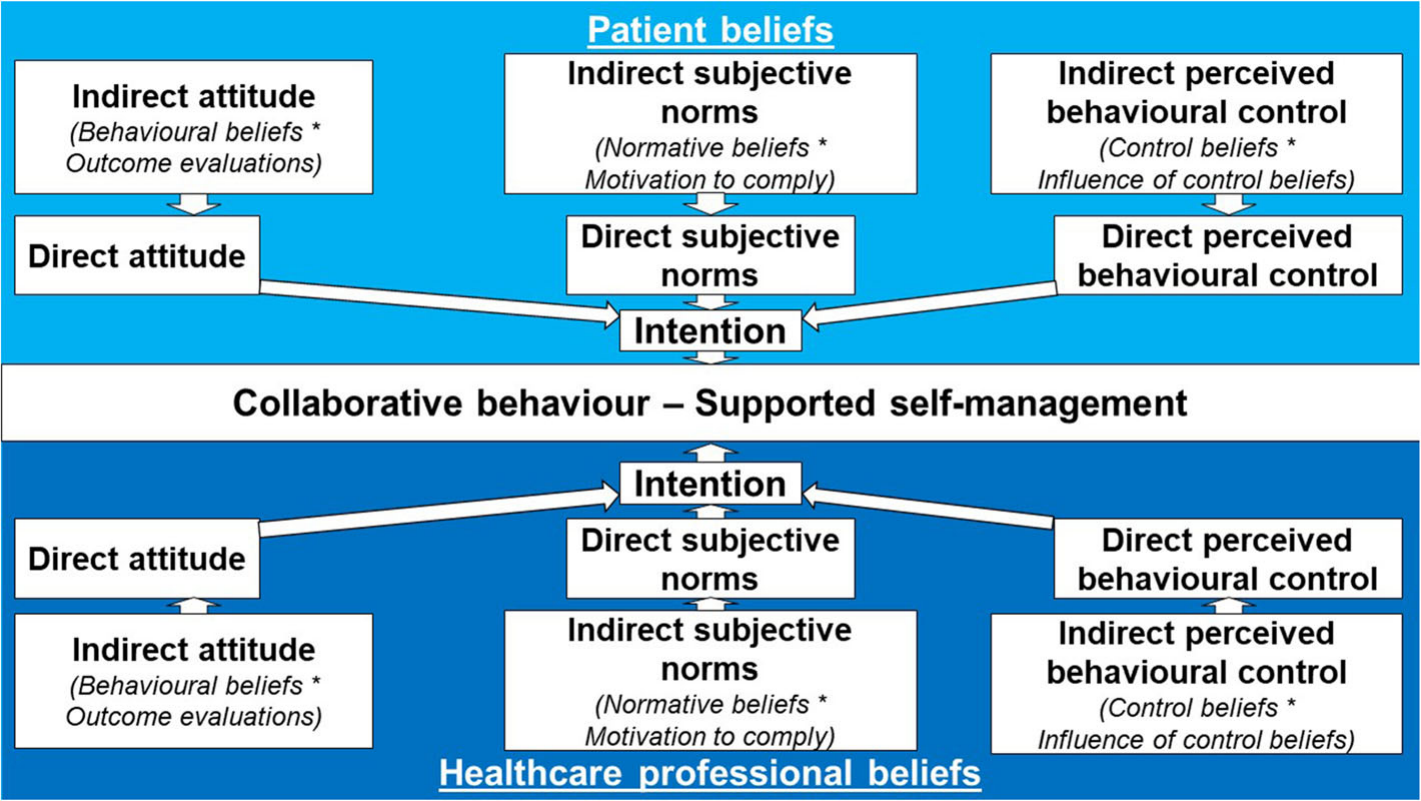
Adapted Theory of Planned Behaviour for Supported Self-Management. Graphical representation (developed by NA) of how the Theory of Planned behaviour applies to collaborative behaviours which require beliefs and intentions of both patients and healthcare professionals

Despite SSM requiring collaborative processes between patients and HSV, (to the researchers’ knowledge) an evidence-gap exists for HSV staff beliefs towards SSM. The *Adapted TPB Model for Collaborative SSM* (Fig. 1) demonstrates the importance of identifying and targeting both patient and HSV beliefs and intentions as involvement of both is required for SSM implementation. Therefore, the mixed-methods exploratory research project sought to use the TPB questionnaire development guide [86] to provide an initial exploration into HSV staff beliefs and intentions towards implementing SSM in LTC healthcare within a single UK local government authority region.

## Methods

The mixed-methods research project was conducted in two phases based on Francis et al.’s [86] guide: (1) qualitative focus group of HSV staff from an existing SSM for LTC project (Additional file 1); (2) quantitative online questionnaire of general LTC staff (Additional file 2).

### Phase 1: focus group

#### Aim

Assess beliefs towards SSM healthcare approach for LTCs in HSV staff with direct and/or indirect involvement in an existing *House of Care*-based project [48].

#### Participants & procedure

HSV staff with direct (patient contact) and/or indirect (service level) involvement in an existing regional SSM for LTCs project were eligible. No exclusions were placed upon age, gender, race, organization, profession, type of patient contact, project involvement, time in current role, time working with LTCs, or experience of SSM. All 11 HSV project staff members were identified and determined by the SSM Project Manager as satisfying the eligibility criteria and were invited to participate. During the focus group, the behavioural focus of *‘HSV staff use of a SSM approach with LTC patients during consultations’* was explored through indirect TPB-based beliefs [86–89]. Six eligible staff members volunteered to participate (2 unavailable, 3 unspecified). Participants were representative of the overall SSM project team (100% female/white/British/≥ 18 years-old/≥ 10 years working with LTC), and comprised a range of organisations (50% NHS, 33% local government, 17% voluntary) and patient contact (50% direct, 50% indirect). NA facilitated, audio recorded and transcribed [90] the 80 m:58 s focus group session. A combination of inductive and deductive thematic analysis was conducted through using a TPB-based researcher-developed coding criterion and inter-rater coding to interpret transcripts in rich detail directly from participants’ utterances [91, 92].

#### Measures & data analysis

TPB-based belief utterances were manually coded onto the Microsoft Office Word 2007™ transcript using the researcher-developed (NA; GO) TPB coding criteria (Table 1) [77, 78, 86]. The criteria was developed using TPB theme and sub-theme definitions, and authors’ understanding of SSM literature. This required identification of potential TPB-based utterances before determining the belief and sub-belief category, and subsequently assigning a specific descriptor and code for each utterance. No minimum or maximum criterion was set for the number of words required to be coded, with potential code durations ranging from a single word to multiple sentences. As belief strength was inferred from utterance frequency (rather than duration), once a code was assigned it was not coded again until the same participant uttered a different belief or a different participant uttered any belief. Subsequently, inter-rater coding was conducted (between NA’s and GO’s codes), code frequencies calculated, and the most frequent 75% of codes used as representations of each respective belief.

**Table 1:** Theory of Planned Behaviour-Based Coding Criteria

Belief Themes		*Belief Sub-Themes*		
Terminology	*Definition*	*Terminology*	*Definition*	*Example*
*Behavioural*	*Belief about consequences of conducting SSM.*	*Instrumental.*	*Beliefs about what SSM will achieve.*	“SSM will *improve/hinder* patient outcomes”.
		*Experiential.*	*Beliefs about how SSM feels to conduct.*	“SSM feels like it *will/won’t* be positive to do”.
*Subjective Norm*	*Beliefs about important others (*e.g. *patients, senior clinicians) beliefs’ towards SSM.*	*Norms.*	*Beliefs about whether SSM is organizationally standard practice.*	“SSM is *not/ promoted* by the organization”.
		*Pressure.*	*Beliefs about how others who are important to them feel about SSM.*	“*All/none* of my colleagues think SSM is positive”.
*Perceived Behavioural Control*	*Beliefs about control over conducting SSM.*	*Self-Efficacy.*	*Beliefs about confidence one can appropriately use SSM.*	“I feel like I am *not/ already able* to use SSM”.
		*Controllability.*	*Beliefs about whether using SSM is their choice.*	“SSM use is not/up to me and the patient”.

### Phase 2: online questionnaire of LTC staff

#### Aim

Assess beliefs towards adopting SSM healthcare approach for LTCs in general HSV staff.

#### Participants & procedure

Researchers developed a questionnaire (Additional file 2) based on *Phase 1* TPB-based beliefs, comprising 51-items across 4 overarching variables: *Demographics*, *Intention, Beliefs and Optional Feedback*. This was published online using SurveyMonkey Gold©, before being piloted and deemed suitable by 5 HSV staff with no formal experience of a SSM project. Staff were approximately representative of regional LTC healthcare (60% female; 100% white/British/≥ 18 years-old), comprised a range of organizations (60% NHS, 40% local government) and patient contact (60% direct, 40% indirect), and all had over 10 years’ experience of LTC.

HSV staff working with LTCs in any capacity were eligible to participate, with no exclusions placed upon age, gender, race, organization, profession, type of patient contact, project involvement, time in current role, time working with LTCs, or experience of SSM. Two complimentary recruitment methods were utilized. First, a senior healthcare line manager directly e-mailed 35 senior, regional HSV line managers to request the further dissemination of an e-mail invitation to participate. Second, regional HSV communications departments disseminated the questionnaire to wider HSV staff internally. As participants were able to omit responses to specific questions, the proportion of the questionnaire completed varied. However, in order to conduct regression analyses responses were required for all sub-variables. Therefore, participants were classed as *Completers* if they omitted a maximum of 1 response to each sub-variable prior to imputation. As a consequence, 115 participants (Table 2) were separated into three completion level groups (*Demographic-Only: n = 20; Demographic & Intention: n = 37; Completers: n = 58*) and demographic differences between completion-level groups assessed prior to *Completer* group regressions. Chi-square tests demonstrated no demographic differences based on survey completion-level (*Demographics-Only; Demographics & Intention; Completers*) for age, gender, organization, service, and time within current role (*p* > .05). However, completers demonstrated significantly greater LTC patient contact (Chi^2^ (8) = 25.196, *p* = .001), and experience both of LTC (Chi^2^ (8) = 16.946, *p* = .031) and SSM (Chi^2^ (10) = 25.812, p = .001).

**Table 2:** Participant Demographic Information

Demographic Information		Participants	
Category	Response Options	Overall (*n* = 115)	Completers (*n* = 58)
Age	18-24y	3%	4%
	25-39y	15%	10%
	40-60y	76%	83%
	>60y	6%	3%
Gender	Female	80%	78%
	Male	16%	17%
	No response	4%	5%
Organisation	NHS	59%	60%
	Council	28%	24%
	Voluntary	8%	9%
	Multiple	2%	4%
	Other	1%	0%
	No response	2%	3%
Service	Primary care	21%	26%
	Secondary care	17%	19%
	Community	42%	34%
	Multiple	13%	9%
	Other	5%	12%
	No response	2%	0%
LTC patient contact	Direct	38%	38%
	Indirect	9%	3%
	Direct & indirect	49%	57%
	None	3%	0%
	No response	1%	2%
Time in role	<1y	9%	14%
	2-5y	23%	22%
	6-9y	19%	17%
	>10y	46%	47%
	No response	3%	0%
Time working with LTC	<1y	5%	5%
	2-5y	12%	10%
	6-9y	13%	12%
	>10y	64%	71%
	No response	6%	2%
SSM involved in current role	Never	22%	12%
	Sometimes	39%	31%
	Often	25%	36%
	Always	12%	19%
	No response	2%	2%

Data were collated and analysed using IBM SPSS Statistics 23™. Data screening, scoring, imputation, internal consistency and variable computation procedures were conducted in accordance with Francis et al.’s [86] procedures. First, individual item responses were scored using either a unipolar scale of 1–7 for concepts which uni-directional measurement was appropriate (e.g. probability), or a bipolar scale of ±3 for concepts which bi-directional measurement was appropriate (e.g. evaluation). Lower scores (e.g. 1 or − 3) reflected *‘Strong Negative Beliefs’* towards SSM, mid-range scores (e.g. 4 or 0) reflected ‘*Neutral’* beliefs with no negative or positive appraisal of SSM, and higher scores (e.g. 7 or + 3) reflected ‘*Strong Positive Beliefs’* towards SSM. Second, where ≤5% data is missing effects are deemed negligible and no single imputation approach is most effective [93–98]. Therefore, item-average data imputation was conducted for the missing 0.37% of intention or belief responses. Third, internal consistency was analysed and specific items removed from sub-variables to maximise internal consistency. Fourth, composite variable scores were calculated and interpreted in accordance with Francis et al.’s procedure [86]. For direct beliefs, SPSS was used to ‘compute’ composite scores for direct measures. For indirect beliefs, each indirect belief sub-domain (e.g. behavioural belief question 1) was multiplied by its respective indirect belief sub-domain (e.g. outcome evaluation question 1), before all weighted belief scores were summed to create a composite belief score (e.g. indirect attitude). Finally, regression analyses were conducted on the *‘Completer’* sample of participants who satisfied the aforementioned questionnaire completion criteria in order to determine whether beliefs significantly associated with intention to implement SSM.

#### Measures & data analysis

The *Completer* sample (*n* = 58) data was analysed using IBM SPSS Statistics 23™ with two multiple regressions to determine whether direct and indirect beliefs (independently) associated with intention to implement SSM, and three (independent) linear regressions to determine whether indirect beliefs associated with their respective direct belief (Additional file 3).

## Results

### Phase 1: focus group

Following an initial 94% belief utterance and 100% code agreement between researchers, 82 utterances across 20 belief codes were agreed upon and the most representative beliefs determined based on utterance frequency *(*Table 3). Beliefs indicated SSM:

1. Improves holistic healthcare provision, communication channels and demands, but requires staff to be supported through simplified organizational pathways to be effective.
2. Effectively applied in other healthcare settings but patients may not always want or understand SSM. Furthermore, to be effective SSM must be tailored to specific healthcare settings and receive senior clinician buy-in (especially GPs in Primary Care settings).
3. Implementation limited by healthcare policy, staff engagement, and in particular resource investments. Healthcare culture-specific training which is tailored specifically to staff’s knowledge, skills and experience is required.

**Table 3:** Theory of Planned Behaviour-Based Beliefs - Focus Group Sample

Belief				Utterance Frequency		Phase 2?
Category	Sub-Belief	Code	Description	No.	Rank	(Yes/No)
Behavioural	Instrumental	BB1	SSM requires support from HSV staff in order to be effective.	1	7	NO
		BB2	Additional/simplified organizational pathways are required in order for SSM to achieve positive outcomes.	5	2	YES
		BB3	SSM improves communication channels.	4	3	YES
		BB4	SSM improves holistic healthcare provision.	7	1	YES
		BB5	SSM improves patient outcomes.	2	5	NO
		BB7	SSM reduces healthcare time demands.	2	5	YES
	Experiential	BB6	SSM is not possible if staff are not supported and facilitated to use it.	3	4	YES
Subjective Norm	Norms	NB1	SSM is effectively being applied in other areas/regions.	2	3	YES
		NB2	SSM is promoted by HSV policy and documentation.	1	6	NO
		NB3	Patients may not always understand, or want staff to implement, SSM healthcare.	7	1	YES
		NB4	Widespread use of SSM would be required in order for it be effectively adopted.	1	6	NO
		NB5	SSM must factor in cultural/ local norms of different HSV settings to be effective.	2	3	YES
		NB7	Patients want to be involved and understand their medication regimens.	2	3	YES
	Pressure	NB6	Without GP buy-in the implementation of a SSM approach is not possible.	5	2	YES
Perceived Behavioural Control	Self-Efficacy	CB1	SSM requires effective co-produced healthcare.	1	5	NO
		CB6	SSM training must be tailored to staff knowledge, skills, experience and needs.	8	3	YES
	Controllability	CB2	SSM is limited by HSV policy and capacity.	9	2	YES
		CB3	Resource investments are required to increase staff SSM control.	13	1	YES
		CB4	SSM requires increased staff engagement to enhance control.	6	4	YES
		CB5	IT/communication sharing improvements are required to enhance staff control.	1	5	NO

### Phase 2: online questionnaire of LTC staff

**Fig. 2:**
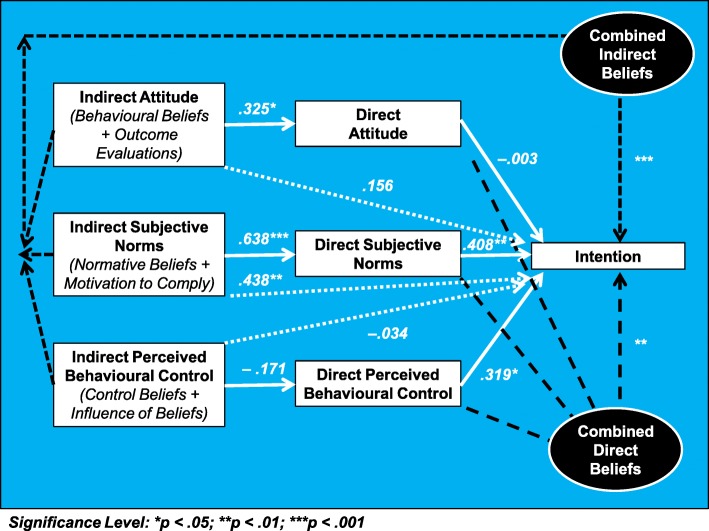
Regression Analyses Summary. Graphical representation (developed by NA) of how the regression analyses conducted on the *Completer* sample relate to the components of the Theory of Planned Behaviour

The 58 ‘*Completers’* (Table 2*)* demonstrated moderate-strength positive attitude beliefs, and weak-strength positive intention, subjective norm, and perceived behavioural control beliefs (Table 4). Figure 2 represents the significance and effect sizes analyses outlined by the TPB questionnaire development guide [86]. This includes two independent multiple regression analyses conducted to determine the associations of overall direct and indirect beliefs with intention, and three independent linear regression analyses conducted to determine the associations of indirect beliefs with their respective direct belief.

**Table 4:** ‘*Completer’* Sample Theory of Planned Behaviour-Based Descriptive Statistics

Component	Intention	Attitude		Subjective Norm		Perceived Behavioural Control	
	Generalised Mean	Direct Mean	Indirect Sum	Direct Mean	Indirect Sum	Direct Mean	Indirect Sum
n	58.	58.	58.	58.	58.	58.	58.
Mean	5.155.	5.868.	49.560.	4.922.	19.569.	4.922.	21.839.
SD	1.832.	1.292.	33.055.	1.363.	21.186.	1.217.	129.00.
Standardized Mean (1–7)	5.155	5.868	5.565	4.922	4.152	4.922	4.401
Interpretation	Weak-Strength Positive Belief.	Moderate-Strength Positive Belief.	Moderate-Strength Positive Belief.	Weak-Strength Positive Belief.	Weak-Strength Positive Belief.	Weak-Strength Positive Belief.	Weak-Strength Positive Belief.

Interpretation of Standardized Mean – Favourability, *Less than 4 = Negative; 4 = Neutral; Greater than 4 = Positive*

Interpretation of Standardized Mean – Strength, *3–5 = Weak; 2–3 or 5–6 Moderate; 1–2 or 6–7 = Strong*

The *‘Combined Direct Beliefs’* multiple regression model significantly explained 37.2% of variance in ‘*Intention’* to conduct SSM with LTCs (F (3, 53) = 10.482, *p* < .001, R^2^ = .372, R^2^_Adjusted_ = .337), with intention significantly explained by the individual predictor variables of ‘*Direct Subjective Norms*’ (β = .408, t (53) = 3.122, *p* = .003) and ‘*Direct Perceived Behavioural Control’* (β = .319, t (53) = 2.585, *p* = .013), but not ‘*Direct Attitude’* (β = −.003, t (53) = −.024, *p* = .981). *‘Combined Indirect Beliefs’* significantly explained 24.7% of variance in ‘*Intention’* (F (3, 54) = 5.893, *p* = .001, R^2^ = .247, R^2^_Adjusted_ = .205), with *‘Intention’* significantly associated with ‘*Indirect Subjective Norms*’ (β = .438, t (54) = 3.460, p = .001), but not ‘*Indirect Attitude’* (β = .156, t (54) = .951, *p* = .346) or ‘*Indirect Perceived Behavioural Control’* (β = −.034, t (54) = −.213, *p* = .832). Linear regression models demonstrated that ‘*Indirect Attitude*’ significantly explained 10.6% of variance in ‘*Direct Attitude’* (F (1, 55) = 6.516, p = .013, R^2^ = .106, R^2^_Adjusted_ = .090), and *‘Indirect Subjective Norms’* significantly explained 40.6% of variance in ‘*Direct Subjective Norms’* (F (1, 56) = 38.348, *p* < .001, R^2^ = .406, R^2^_Adjusted_ = .396). However, ‘*Indirect Perceived Behavioural Control*’ non-significantly explained 2.9% of variance in ‘*Direct Perceived Behavioural Control’* (F (1, 55) = 1.652, *p* = .204, R^2^ = .029, R^2^_Adjusted_ = .012).

## Discussion

Research previously explored the relative strengths and challenges of SSM [35], and the patient, communicative, and organizational components associated with enhance outcomes [2, 6, 33, 40–45, 60–73]. However, despite chronic conditions models [5, 32, 34] which form the foundation of SSM placing emphasis on beliefs being fundamental to behaviour, and health psychology theory demonstrating that intention is associated with actual behaviour [77, 78], staff intentions and beliefs towards SSM have received little research attention. As the *Adapted TPB for SSM Model* (Fig. 1) demonstrates, this means that a significant part of the picture of what is required in order for SSM to be effectively implemented is currently unknown. Therefore, the mixed-methods research project sought to provide an initial exploration into a SSM research gap by determining the likelihood of SSM being implemented in a rural region where SSM is promoted by assessing HSV beliefs towards SSM and which beliefs are associated with intention towards implementing this approach.

The focus group demonstrated staff from an existing SSM project believe that SSM has positive implications for healthcare outcomes, but is unlikely to be effective unless enhancements are made to organizational policy, resources, senior clinician buy-in, and healthcare culture-specific training. The online questionnaire also demonstrated that general LTC staff believe that SSM has positive implications for healthcare outcomes. However, staff were unlikely to implement SSM due beliefs that they had limited ability or control over attempting this approach, and that there was limited social encouragement towards it. Crucially, staff attitudes towards whether SSM was beneficial or achieved positive outcomes did not explain intention towards implementing it. However, if staff did not feel important others (such as GPs in primary care settings) supported SSM and that they had control over using it, or they did not receive positive social encouragement towards it, staff were unlikely to intend to implement SSM.

As aforementioned, attitudes towards whether SSM achieves positive outcomes or whether they were in favour of using it did not explain intentions towards it, which may have two potential implications which require further research. First, if attitudes towards SSM do not explain intention towards implementing it then healthcare initiatives which focus on enhancing attitudes are unlikely to be effective, and focusing on alternative beliefs may be a more appropriate use of time and resources. Alternatively, as both focus group and online questionnaire samples demonstrated positive attitudes towards SSM, this possibly reflects that focusing on enhancing attitudes for those with pre-existing positive attitudes is unlikely to result in a significant enhancement. Therefore, future research should sample those with a greater range of views about whether SSM (if effectively conducted) has positive outcomes, in order to guide the degree to which attitudes influence SSM implementation.

While attitude did not associate with intention, intention towards SSM was significantly explained by beliefs for whether important others supported SSM implementation and perceived control over whether or not it should be implemented. This has two key implications for current issues which could potentially be used as opportunities to target and enhance intention towards SSM via social norm and control beliefs. First, participants had less intention to implement SSM if they perceived patients did not understand or want this approach, senior clinicians did not support it, or that SSM had not been tailored to the specific healthcare culture. However, participants believed that SSM had been effectively applied in other healthcare context. As research indicates that patients generally support SSM [2, 32], including patients in SSM initiatives to provide their direct experiences and perspectives may potentially enhance staff intentions towards SSM. Additionally, as staff perceived that SSM had been effectively implemented in other healthcare settings, including staff from other successful projects to prove information and assist the development of tailored programmes may also enhance intention. Second, participants demonstrated lower intentions towards SSM if they perceived they had low control or ability to implement SSM, which stemmed from beliefs that organizational policy and culture-specific training enhancements were required to increase engagement. Similarly, learning from previous successful programmes may also have a positive impact upon intention towards SSM. Hence, social and control beliefs significantly associated with intention towards SSM, and provide an opportunity to enhance SSM implementation through including patients and stakeholders from previous successful SSM programmes to enhance components believed to be confounds for successful implementation.

Based on TPB principles it would be expected that indirect beliefs would only associate with intention via direct beliefs. However, contrary to these principles, indirect social beliefs significantly directly associated with intention, indicating that if one perceives sufficient social encouragement towards SSM then they are significantly more likely to intend to implement SSM (independent of all other beliefs). This provides promise for SSM healthcare research and practice as it potentially indicates that, if appropriate consideration is given to promoting and supporting staff to feel encouraged to implement SSM, staff are significantly more likely to intend to do so. However, this raises an ethical issue as increasing social pressure in this context requires encouraging staff in a positive, supportive manner to promote SSM as social normative and supported by organizations, rather than negatively pressuring staff that negative consequences will occur if they are unable to implement SSM. Hence, social encouragement towards conducting SSM significantly associated with intention towards implementing this approach, and future research should seek to determine how this may be optimally achieved.

To the researchers’ knowledge no previous research has assessed HSV staff beliefs towards SSM. As intention is associated with actual behaviour [77, 78], this exploratory study sought to provide an initial investigation into a key element of SSM and determine beliefs that may be used for increasing intention towards implementing SSM. However, the study only provided a starting point for psychological research into staff beliefs towards SSM for LTCs, with key considerations required. First, the study sought to determine beliefs that associate with intention to conduct SSM, which in turn is believed to associate with actual behaviour. Furthermore, as previous studies have assessed patient beliefs towards SSM, the study only explored staff beliefs towards implementing SSM. However, future studies should incorporate both patient and HSV beliefs in addition to measures of actual behavioural changes pre- and post-implementation of SSM programmes to determine actual behaviour in accordance with the *Adapted TPB for SSM Model* (Fig. 1). Second, while the TPB provided a useful framework for determining intention towards implementing SSM, direct and indirect beliefs only explained 37.2 and 24.7% of variance in intention respectively. Therefore, future research should determine the extent to which beliefs included in alternative health psychology models [76], and external constructs relating to patient and organisational barriers and facilitators, influence intention. Hence, this study provided a useful exploration into staff beliefs towards implementing SSM with LTCs patients and highlighted areas of potential focus for enhancing intentions towards implementing SSM, but future research is required to build upon these exploratory findings.

### Strengths & limitations

To the researchers’ knowledge, the exploratory project was the first to use psychological principles that underlie chronic care models to assess staff beliefs towards SSM [2, 5, 32, 34, 62, 64–72]. The TPB was selected due to having a strong pre-existing research base for assessing beliefs towards various health behaviours combined with having a pre-existing, validated questionnaire development guide [80, 81, 86, 99, 100]. However, within health psychology there is ongoing debate on whether issues with parsimony, predictive validity and utility mean that the TPB should be ‘retired’ in favour of alternative models, or whether aforementioned issues stem from misunderstanding TPB components and research [77–79]. As highlighted by Sniehotta et al.*’s* [79] comprehensive critique of TPB, while *Intention* and *Perceived Behavioural Control* are relatively consistent predictors of behaviour and interventions targeting intention are likely to result in behaviour change, a significant confound of the TPB is the *‘Intention-Behaviour Gap’* which categorises discrepancies between these constructs which cannot be accounted for by TPB components alone and is a key area of ongoing research. Therefore, the approach adopted provided an established, evidence-based psychological framework for exploring and assessing a SSM research gap. However, future research should consider the efficacy of trialling and contrasting alternative models and frameworks [76] to determine the psychological constructs most relevant to SSM.

The TPB questionnaire development guide ensured beliefs were representative of regional healthcare cultures across a range of organisations and roles, including those with no formal SSM experience. While mixed-methods studies may be more complex, time, and resource intensive than quantitative or qualitative methods individually, they may offset the weaknesses of each approach through combining inductive and deductive reasoning to assess both observational and statistical information, and reduce potential researcher-biases [101–103]. However, construct validity issues were present which may reflect questionnaire development guide and/or measure construction confounds. First, to promote coherence of focus group discussions, TPB-based beliefs were indirectly explored through questioning the facilitators and barrier of an existing SSM project. However, direct questioning of specific TPB-based beliefs may potentially generate a more focussed exploration of beliefs. Second, pilot feedback indicated that the questionnaire was representative of intended beliefs, but may benefit from containing less questions. As TPB development guide requirements meant this was not possible and only 50.4% participants completed the questionnaire, a more flexible approach to questionnaire development may be beneficial. Finally, specific variables were removed from composite scores to improve internal consistency. However, as one belief was ultimately only represented by two sub-variables, this may indicate that specific questions may not have been optimally representative of intended beliefs and/or were sub-optimally constructed. Hence, the TPB questionnaire development guide provided a useful framework belief but future research should build upon construct confounds to improve validity.

The small, single rural geographical region where healthcare is integrated and SSM is promoted was a relevant research setting, but may have raised generalisability and recruitment confounds. First, while different SSM programmes have achieved positive outcomes across cultures [40–45], rural settings experience the dual challenge of increasingly elderly populations with higher LTC levels and recruitment difficulties compared to urban settings [104]. This may influence staff beliefs and consequently intervention implementation requirements. Second, to enhance recruitment a combination of intranet and senior HSV stakeholder e-mail invitation approaches were used. However, as existing IT mechanisms cannot accurately determine who disseminated or accessed the questionnaire, recruitment may have been confounded by senior stakeholders’ personal beliefs towards SSM reducing dissemination and/or potential participants’ perceptions of lacking time or capacity. Therefore, it is uncertain whether greater questionnaire completion levels by community than hospital staff was due to a lack of awareness, engagement or time for either the questionnaire or SSM in general. Hence, future research may benefit from conducting a cross-regional approach, early targeting and engagement of patients and senior stakeholders, and supplementary recruitment methods to maximise dissemination and completion. For example, involving Patient & Public Involvement or HSV management groups in the design and recruitment processes, and visiting staff on hospital wards to explain the project and provide hard copies of questionnaires.

Despite attempts to promote participation from a range of SSM and LTCs experience, two key implications are present from the *Completer* sample being smaller than desired and having high levels of patient contact and both SSM and LTC experience. First, experience levels appear to have influenced perceived confidence, motivation and/or importance of participating but, as the first exploration of staff beliefs towards implementing SSM, uncertainty remains for the generalisability of findings and whether greater experience of LTCs and/or SSM enhances or inhibits beliefs towards implementing SSM. Second, only 50.4% of 115 participants met the questionnaire completion criterion, which has implications for ethical requirements and analyses power. As ethical requirements ensured participants could omit responses to questions but in order to also satisfy regression requirements a strict completion criterion was set. This raised a significant confound that the *Completer* sample of 58 participants was adequately powered for linear but not multiple regressions, and consequently results must be interpreted as an exploratory foundation for future research to replicate and develop upon, rather than definitive findings. Therefore, while the experienced sample provided valuable information, future replications comprising a larger sample with a greater range of experience is required to increase generalisability and reliability.

Finally, a key strength was the study rationale. Despite comprising different components, chronic care (and consequently SSM) models are fundamentally based on psychological theory [5, 6, 32, 34, 49]. However, despite all models proposing that SSM encompasses a combination of patient, professional, communication, and organizational factors, SSM implementation has typically occurred in the absence of research to determine staff beliefs towards SSM. Therefore, despite active attempts to enhance SSM implementation and outcomes, through neglecting HSV beliefs a significant component required to effectively implement SSM is unknown. Hence, while future research is required to build upon aforementioned strengths and improve limitations, the study highlights the importance of not only assessing and improving patient and organizational outcomes, but also HSV SSM beliefs and subsequently behaviours.

## Conclusions

Despite SSM requiring collaborative behaviours between patients and clinicians, and health psychology theory which underpins SSM proposing beliefs are critical to behaviours, little research has assessed healthcare professionals' beliefs towards SSM. The findings of the exploratory study indicate that staff experienced in both SSM and LTC demonstrate moderate-strength beliefs that SSM improves holistic healthcare outcomes. However, in spite of positive attitudes, staff demonstrated weak-strength intentions to implement SSM, and weak-strength beliefs that SSM is socially encouraged, promoted by important others, and that they had the perceived control and ability to implement it. Additionally, intentions towards conducting SSM was only associated with beliefs about whether they had the choice and ability to actually implement it, or whether SSM was socially encouraged. Therefore, while staff believe that SSM improves outcomes, future healthcare research and provision is required which targets and enhances the aforementioned beliefs (as well as organizational and external factors influencing these beliefs) to increase SSM implementation.

## Additional files

Additional file 1: Evaluation of perceptions of shared-management provision for those with long-term conditions in NHS borders. (PDF 435 kb)

Additional file 2: Supported self-management for people living with long-term conditions. (PDF 899 kb)

Additional file 3: Statistical analyses. (DOCX 12 kb)

## Data Availability

The datasets used and/or analysed during the current study are available from the corresponding author on reasonable request.

## Abbreviations

HBM: Health belief model
HSV: Health, Social Care and Voluntary
LTC: Long-term conditions
PCC: Person-Centred Care
SSM: Supported self-management
TPB: Theory of planned behaviour
