# Supplementary material for: “It all needs to be a full jigsaw, not just bits”: exploration of healthcare professionals’ beliefs towards supported self-management for long-term conditions
Source: BMC Psychol. 2019 Jun 24;7:38. doi: 10.1186/s40359-019-0319-7 (PMC6591939; doi:10.1186/s40359-019-0319-7)
Supplement: Supplementary file 1 — Evaluation of perceptions of shared-management provision for those with long-term conditions in NHS borders. (PDF 435 kb) [file 40359_2019_319_MOESM1_ESM.pdf]

Shared-Management for Long-Term Conditions

Focus Group Facilitation Schedule

PROJECT TITLE: Evaluation of Perceptions of Shared-Management Provision for those with Long-Term Conditions in NHS Borders

General Reminders

- It is okay to asking ‘probing questions’ if people are struggling to answer
  - E.g. “Would you mind explaining/elaborating on this further please?”
- Fine to provide short responses but avoid affirmations of responses as this may lead discussions
- Ensure that discussions are controlled so that everyone has an opportunity to speak
- Potentially important elements that may be raised that I should look out for are:
  - (1) House of Care model (2) Green pathway (3) Care plan and audit (3) BRC referrals (5) Practice contact audit (5) Shared management and stability audit

Preliminary Tasks

- Set up the Audio recorder and all documentation
- Welcome everyone to the room, provide documentation and sticky labels (for name badges)
- Provide a brief explanation of the information sheet and consent form, and ask them to sign it
  - Mention that if anybody is unhappy with any of the elements then they are free to leave. However, we hope they are happy to participate as their contribution is extremely valuable

Welcome

- Welcome everyone to the sessions and thank them for coming along
- Introduce who I am, my role and the purpose of the session
- Explain why everyone was invited and the goal of the setting
- Explain group guidelines
  - (1) No right or wrong answers so please be respectful of everyone else’s opinions
  - (2) Audio recording so please speak one person at a time
  - (3) First names only (if everyone is okay with this)
  - (4) Purpose is to find out a range of opinions (and not necessarily to agree with each other) so please listen to what everyone else has to say
  - (5) Phones/pagers on silent if possible please
  - (6) My role is the moderator so please direct conversation to each other rather than me
- Check for agreement, turn on the audio recorder and proceed

Opening Question [Maximum of 5 minutes]

1. In order to ensure that we all know each other could we please all go around the table and state:
   a. (i) Name (ii) Organisation/Role (iii) Role within the project

Introductory Question [Maximum of 10 minutes]

The project aimed to support shared-management of Long-Term Conditions in older adults

1. Please discuss what your perception of the importance of shared-management for Long-Term Conditions is, and whether there has been any change since the beginning of the project

Shared-Management for Long-Term Conditions

Transition Question [Maximum of 10 minutes]

The House of Care model was integral to the project and a copy of this has been provided to you all

1. How important and relevant do you think the model, and its components, were for utilising a shared-management approach?

Key Questions [Maximum of 40 minutes]

Next, I would like us to think about the current project for supporting shared-management for older adults with Long-Term Conditions. In terms of the existing project, what elements do you think specifically

1. Contributed to the success of the project?
2. Were ineffective elements of the project?

One purpose of the project was to gain information in order to assist the development of a larger, locality shared-management project for Long-Term Conditions in all adults. Having thought about what worked well, in terms of the development of a larger scale project what elements do you think specifically

1. Would not be beneficial to include in future work? Particularly in relation to applying this for all adults
2. Would be critical elements to include in future work? In relation to applying this for all adults

Ending Question [Maximum of 10 minutes]

1. All things considered, of what we discussed what would you say was the most important?

Summary Questions [Maximum of 10 minutes]

Provide a summary of the key points of the session

1. Does this sound like a fair reflection of what has been said and the key points?
2. Is there anything that we have missed that anyone would like to raise?

Closing the Session [Maximum of 5 minutes]

Thank everyone for coming and ask them to complete the questionnaire
