# Supplementary material for: “It all needs to be a full jigsaw, not just bits”: exploration of healthcare professionals’ beliefs towards supported self-management for long-term conditions
Source: BMC Psychol. 2019 Jun 24;7:38. doi: 10.1186/s40359-019-0319-7 (PMC6591939; doi:10.1186/s40359-019-0319-7)
Supplement: Supplementary file 2 — Supported self-management for people living with long-term conditions. (PDF 899 kb) [file 40359_2019_319_MOESM2_ESM.pdf]

Supported Self-Management for People Living with Long-Term Conditions

Demographic Information

Supported Self-Management Definition:

A supported self-management (SSM) approach to health and social care for people living with long-term conditions (LTC) aims to *support and enable* people to make *positive changes* to their behaviour in order to *support and promote health and well-being*. SSM and self-management differ in that:

- Self-management aims to enable people to take responsibility for their own behaviour
- SSM aims to promote health and social care professionals, teams and services to use their knowledge and skills to ensure that people living with LTC have the knowledge, confidence, skills and support to manage their condition(s).

Crucially, SSM involves building upon traditional medical models of care to ensure that a person-centred approach where people are actively involved in their health and social care decision making is implemented.

Survey Information

The survey is an opportunity for Scottish Borders staff who work in Health & Social Care in any discipline in both the statutory and 3rd sector to have their say on SSM in relation to:

- The advantages and disadvantages of SSM
- Whether SSM is usual practice within your organisation
- The ease and difficulty of adopting a SSM approach

The multi-choice questionnaire takes approximately 15 minutes to complete. There are no right or wrong answers, and your responses are completely anonymous, so please feel free to be as honest as possible. As the survey is aimed at understanding staff beliefs' with a view to using this to assist the learning and development of staff within the Scottish Borders, your opinion is extremely valuable and appreciated.

Ethics Information

The *Service Evaluation* was reviewed and approved by NHS Borders Governance on 07.10.2016, in affiliation with the University of St Andrews (from 19.10.2016). By completing the questionnaire you are providing consent to participate in the survey, however, your participation is voluntary, you may omit sections that you do not want to answer, and may withdraw at any time by pressing the 'Exit' button.

Your responses are completely anonymous and confidential, and will be stored on an encrypted, password-protected NHS Borders data stick for up to one year. Anonymous responses shall only be accessible to the Lead Investigator, and the project Supervisors (below) upon request. The results of the evaluation shall be used to guide staff learning and development within the Scottish Borders, to create a report as part of the Lead Investigator's training portfolio, and may be used for publication and conference presentation purposes.

If you have any questions, please feel free to contact:

- Lead Investigator - Niall Anderson - niall.anderson@borders.scot.nhs.uk
- Evaluation Supervisor - Dr Allyson McCollam - allyson.mccollam@borders.scot.nhs.uk
- Research Supervisor - Dr Gozde Ozakinci - go10@st-andrews.ac.uk
- NHS Borders Governance - Joy Dawson - research.governance@borders.scot.nhs.uk

Thanks very much for taking the time to consider participating!

1. What is your age?

- ◯ 17 or younger
- ◯ 18-24 years
- ◯ 25-39 years
- ◯ 40-60 years
- ◯ 61 or older

2. To which gender do you most identify?

- ◯ Female
- ◯ Male
- ◯ Trans
- ◯ Prefer to not answer

3. Which organisation(s) do you work for?

- ☐ NHS
- ☐ Council
- ☐ 3rd Sector
- ☐ Other (please specify)

4. What category of service(s) is your main role within?

- ☐ Primary Care
- ☐ Hospital Services
- ☐ Community Services
- ☐ Other (please specify)

5. What form(s) of contact do you have in your role with people living with LTC?

- ◯ Direct
- ◯ Indirect
- ◯ Both direct and indirect
- ◯ None

6. How long have you been employed within your current role?

- ◯ 1 year or less
- ◯ 2-5 years
- ◯ 6-9 years
- ◯ 10 or more years

7. How long have you worked with people living with LTC?

- ◯ 1 year or less
- ◯ 2-5 years
- ◯ 6-9 years
- ◯ 10 or more years

8. Does your work currently involve using SSM?

- ◯ Never
- ◯ Sometimes
- ◯ Often
- ◯ Always

Intention to Conduct SSM

SSM Definition:
SSM aims to support and enable people living with LTC to make positive changes to their behaviour in order to support and promote health and well-being.

9. Please select one response per statement

	Strongly Disagree	Disagree	Slightly Disagree	Neither Agree or Disagree	Slightly Agree	Agree	Strongly Agree
I *expect* to use SSM with people living with LTC	○	○	○	○	○	○	○
I *want* to use SSM with people living with LTC	○	○	○	○	○	○	○
I *intend* to use SSM with people living with LTC	○	○	○	○	○	○	○

Attitude Towards SSM

SSM Definition:
SSM aims to support and enable people living with LTC to make positive changes to their behaviour in order to support and promote health and well-being.

10. Please select one response per statement

	Strongly Disagree	Disagree	Slightly Disagree	Neither Disagree or Agree	Slightly Agree	Agree	Strongly Agree
Using a SSM approach with people living with LTC is... *Beneficial*	◯	◯	◯	◯	◯	◯	◯
Unpleasant	◯	◯	◯	◯	◯	◯	◯
Unsatisfying	◯	◯	◯	◯	◯	◯	◯
Useful	◯	◯	◯	◯	◯	◯	◯

11. Please select one response per statement

	Strongly Disagree	Disagree	Slightly Disagree	Neither Disagree or Agree	Slightly Agree	Agree	Strongly Agree
Using SSM provides more holistic, person-centred care	◯	◯	◯	◯	◯	◯	◯
SSM would need changes to organisational pathway	◯	◯	◯	◯	◯	◯	◯
SSM improves staff communication channels	◯	◯	◯	◯	◯	◯	◯
Staff would need additional support to conduct SSM	◯	◯	◯	◯	◯	◯	◯
In the long-term, SSM reduces the amount of time I spend with each person living with LTC	◯	◯	◯	◯	◯	◯	◯

12. Please select one response per statement

	Strongly Disagree	Disagree	Slightly Disagree	Neither Disagree or Agree	Slightly Agree	Agree	Strongly Agree
Providing holistic, person-centred care is desirable	◯	◯	◯	◯	◯	◯	◯
Changing organisational pathways is desirable	◯	◯	◯	◯	◯	◯	◯
Improving staff communication channels is desirable	◯	◯	◯	◯	◯	◯	◯
Additional support for staff would be desirable for people living with LTC	◯	◯	◯	◯	◯	◯	◯
Reducing the amount of time I spend with each person is desirable	◯	◯	◯	◯	◯	◯	◯

Social Beliefs Towards SSM

SSM Definition:
SSM aims to support and enable people living with LTC to make positive changes to their behaviour in order to support and promote health and well-being.

13. Please select one response per statement

	Strongly Disagree	Disagree	Slightly Disagree	Neither Disagree or Agree	Slightly Agree	Agree	Strongly Agree
Most colleagues who are important to me think that SSM for people living with LTC is something that I should do	◯	◯	◯	◯	◯	◯	◯
It is expected of me by my organisation that I use SSM with people living with LTC	◯	◯	◯	◯	◯	◯	◯
I feel under social pressure to use SSM with people living with LTC	◯	◯	◯	◯	◯	◯	◯

14. Please select one response per statement

	Strongly Disagree	Disagree	Slightly Disagree	Neither Disagree or Agree	Slightly Agree	Agree	Strongly Agree
People living with LTC think SSM is something that I should do	◯	◯	◯	◯	◯	◯	◯
GPs/Doctors approve of staff using SSM	◯	◯	◯	◯	◯	◯	◯
Most other healthcare organisations currently use SSM	◯	◯	◯	◯	◯	◯	◯
SSM is a normal part of the culture of my organisation	◯	◯	◯	◯	◯	◯	◯
People living with LTC want to have a greater understanding and involvement in their care	◯	◯	◯	◯	◯	◯	◯

15. Please select one response per statement

	Strongly Disagree	Disagree	Slightly Disagree	Neither Disagree or Agree	Slightly Disagree	Agree	Strongly Agree
Ensuring that care fits with what people living with LTC want is important to me	○	○	○	○	○	○	○
GPs/Doctors approval of the approach I adopt is important to me	○	○	○	○	○	○	○
Other healthcare organisations approach to care influences the approach in my organisation	○	○	○	○	○	○	○
Working in a way that fits with the norms of my organisational is important to me	○	○	○	○	○	○	○
Ensuring that the care I provide involves the person living with LTC as much as they would like is important to me	○	○	○	○	○	○	○

Beliefs of Control over SSM

SSM Definition:
SSM aims to support and enable people living with LTC to make positive changes to their behaviour in order to support and promote health and well-being.

16. Please select one response per statement

	Strongly Disagree	Disagree	Slightly Disagree	Neither Disagree or Agree	Slightly Disagree	Agree	Strongly Agree
I am confident that I could use SSM with people living with LTC	○	○	○	○	○	○	○
Using SSM with people living with LTC is easy	○	○	○	○	○	○	○
The decision of whether I use SSM is beyond my control	○	○	○	○	○	○	○
Whether I use SSM or not with people living with LTC is entirely up to me	○	○	○	○	○	○	○

17. Please select one response per statement

	Strongly Disagree	Disagree	Slightly Disagree	Neither Disagree or Agree	Slightly Agree	Agree	Strongly Agree
The organisation would need to invest more resources and time for me to use SSM	○	○	○	○	○	○	○
I am limited in my ability to provide SSM by organisational policy and capacity	○	○	○	○	○	○	○
Training tailored to my knowledge, experience and needs would be required for me to provide SSM	○	○	○	○	○	○	○
Increased staff engagement is required for staff to use SSM	○	○	○	○	○	○	○

18. Please select one response per statement

	Strongly Disagree	Disagree	Slightly Disagree	Neither Disagree or Agree	Slightly Agree	Agree	Strongly Agree
When more resources and time are invested in something I am more likely to do it	○	○	○	○	○	○	○
Changing organisational policy and capacity would increase my capacity to use SSM	○	○	○	○	○	○	○
I would be more likely to use SSM if I received tailored training	○	○	○	○	○	○	○
I would be more likely to use SSM if I was more engaged in it	○	○	○	○	○	○	○

Comments/Feedback

SSM Definition:
SSM aims to support and enable people living with LTC to make positive changes to their behaviour in order to support and promote health and well-being.

19. Please feel free to provide further comments and/or feedback
