# Supplementary material for: “It all needs to be a full jigsaw, not just bits”: exploration of healthcare professionals’ beliefs towards supported self-management for long-term conditions
Source: BMC Psychol. 2019 Jun 24;7:38. doi: 10.1186/s40359-019-0319-7 (PMC6591939; doi:10.1186/s40359-019-0319-7)
Supplement: Supplementary file 3 — Statistical analyses. (DOCX 12 kb) [file 40359_2019_319_MOESM3_ESM.docx]

**Additional File 3:** Statistical Analyses

| **Analysis** | | **Description** |
| --- | --- | --- |
| **No.** | **Component** |  |
| **1** | **Hypothesis** | Completion groups will non-significantly differ in terms of demographic variables. |
|  | **Statistical Analysis** | Chi-Square x 7. |
|  | **Dependent Variable** | Completion level. |
|  | **Independent Variable(s)** | Age; Gender; Organization; Service; LTC patient contact; Time in Role; Time in LTC; Role SSM use. |
|  | **Parametric Assumptions** | All assumptions satisfied. |
| **2** | **Hypothesis** | Combined direct beliefs will significantly predict intention to conduct SSM for LTCs. |
|  | **Statistical Analysis** | Multiple Regression. |
|  | **Dependent Variable** | Mean Intention. |
|  | **Independent Variable(s)** | Mean Direct Attitude; Mean Direct Subjective Norm; Mean Direct Perceived Behavioural Control. |
|  | **Parametric Assumptions** | Outlier assumption violated (Std. Res = -3.405). Participant 10 removed and all assumptions satisfied. |
| **3** | **Hypothesis** | Combined indirect beliefs will non-significantly predict intention to conduct SSM for LTCs. |
|  | **Statistical Analysis** | Multiple Regression. |
|  | **Dependent Variable** | Mean Intention. |
|  | **Independent Variable(s)** | Sum Indirect Attitude; Sum Indirect Subjective Norm; Sum Indirect Perceived Behavioural Control. |
|  | **Parametric Assumptions** | All assumptions satisfied. |
| **4** | **Hypothesis** | Indirect attitude beliefs will significantly predict direct attitudes towards SSM for LTCs. |
|  | **Statistical Analysis** | Linear Regression. |
|  | **Dependent Variable** | Mean Direct Attitude. |
|  | **Independent Variable(s)** | Sum Indirect Attitude. |
|  | **Parametric Assumptions** | Outlier assumption violated (Std. Res = -3.310; Cook’s = 1.554). Participant 38 removed and all assumptions satisfied. |
| **5** | **Hypothesis** | Indirect subjective norm beliefs will significantly predict direct subjective norm beliefs towards SSM for LTCs. |
|  | **Statistical Analysis** | Linear Regression. |
|  | **Dependent Variable** | Mean Direct Subjective Norm. |
|  | **Independent Variable(s)** | Sum Indirect Subjective Norm. |
|  | **Parametric Assumptions** | All assumptions satisfied. |
| **6** | **Hypothesis** | Indirect perceived behavioural control beliefs will significantly predict direct perceived behavioural control beliefs towards SSM for LTCs. |
|  | **Statistical Analysis** | Linear Regression. |
|  | **Dependent Variable** | Mean Direct Perceived Behavioural Control. |
|  | **Independent Variable(s)** | Sum Indirect Perceived Behavioural Control. |
|  | **Parametric Assumptions** | Outlier assumption violated (Std. Res = -3.304). Participant 58 removed and all assumptions satisfied. |
